# Resistance and resilience to experimental gingivitis: a systematic scoping review

**DOI:** 10.1186/s12903-019-0889-z

**Published:** 2019-09-11

**Authors:** Charifa Zemouri, Nicholas S. Jakubovics, Wim Crielaard, Egija Zaura, Michael Dodds, Bettina Schelkle, Bruno G. Loos

**Affiliations:** 1 Department of Preventive Dentistry, Academic Centre for Dentistry Amsterdam (ACTA), University of Amsterdam and Vrije Universiteit Amsterdam, Amsterdam, The Netherlands; 2 Centre for Oral Health Research, School of Dental Sciences, Newcastle University, Framlington Place, Newcastle upon Tyne, UK; 3 Mars-Wrigley Confectionery, Chicago, IL USA; 4 ILSI Europe a.i.s.b.l, Avenue E. Mounier 83 (box 6), Brussels, Belgium; 5 Department of Periodontology, Academic Centre for Dentistry Amsterdam (ACTA), University of Amsterdam and Vrije Universiteit Amsterdam, Amsterdam, The Netherlands

**Keywords:** Experimental gingivitis, Systematic scoping review, Biomarkers, Resistance, Resilience

## Abstract

**Background:**

This systematic scoping review aimed to identify changes in biomarkers of microbiological, immunological and biochemical origin during experimental gingivitis (EG) studies that might indicate resistance and resilience.

**Methods:**

The term ‘experimental gingivitis’ was run in PubMed from inception to April 11th, 2018. From the 411 studies retrieved, 22 studies were included for this review.

**Results:**

Studies reporting data on biomarker changes during and after full mouth EG trial were included. Two studies reported findings on changes in biomarkers of microbiological, 12 on immunological and eight on biochemical origin. Changes were reported in the induction phase, and occasionally in the resolution phase. The microbiological composition of both supragingival and subgingival dental plaque changed over the course of EG to a more pathogenic direction, but showed a shift back to a more normal composition. This indicates resilience of the oral microbiome. For immunological biomarkers, it was challenging to retrieve a robust pattern of changes across multiple studies. IL-1β and IL-6 in saliva and in gingival crevicular fluid increased during induction phase and returned in the resolution phase below baseline values. The biochemical parameters cystatin-SN, cystatin-S and lactoferrin in saliva were increased at the end of induction phase, however also here no clear pattern emerged based on all available studies.

**Conclusions:**

More research is needed to investigate which microbiological, immunological, and biochemical biomarkers can be useful for future investigations into the resistance and resilience of the oral cavity to experimental gingivitis.

**Electronic supplementary material:**

The online version of this article (10.1186/s12903-019-0889-z) contains supplementary material, which is available to authorized users.

## Background

**Fig. 1:**
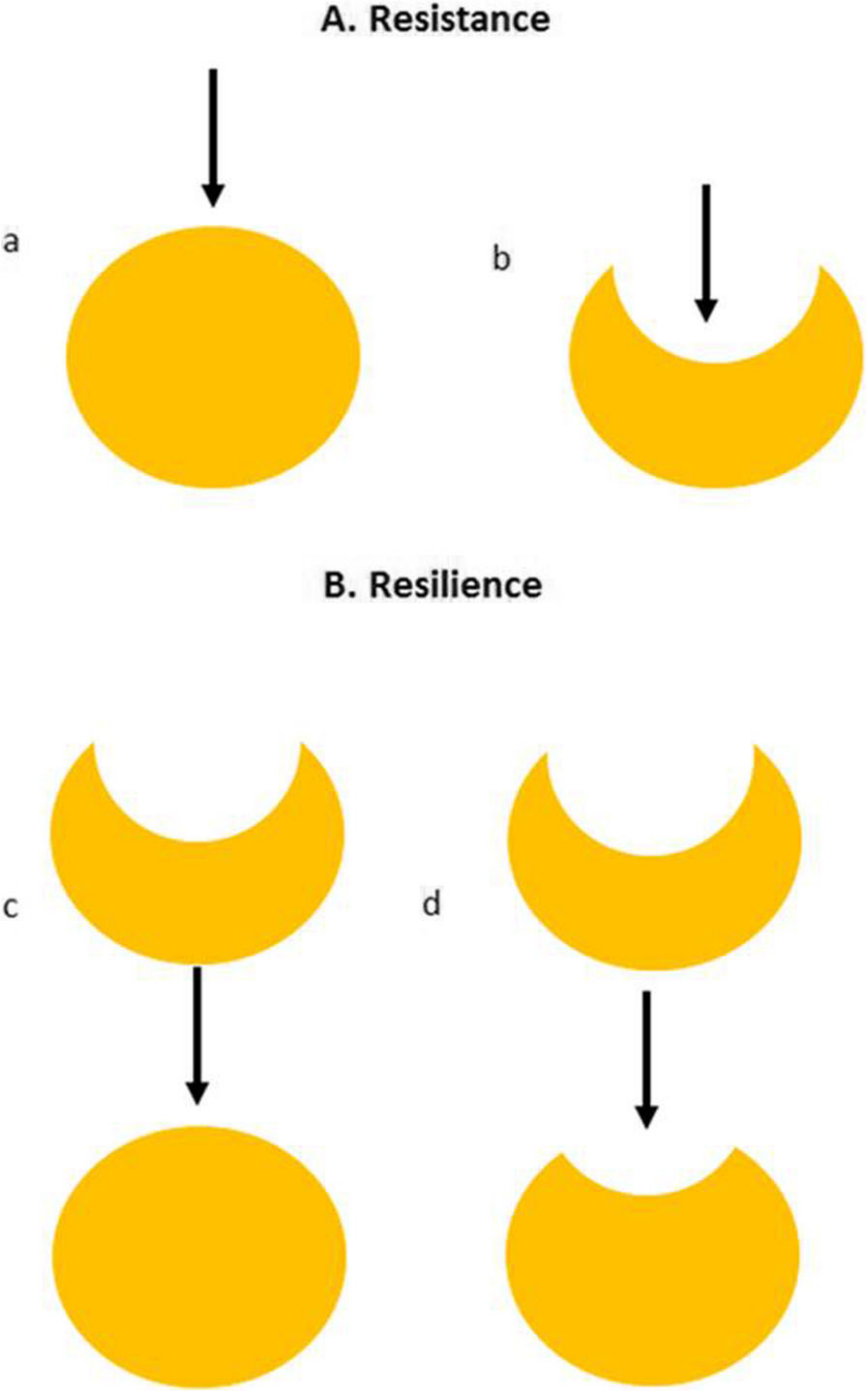
Definitions of resistance and resilience in the current systematic scoping review on EG. In Panel **a** we define resistance, i.e. the ability to remain unchanged during perturbation (arrows in a and b): High resistance is defined as a low response during induction phase (a), while low resistance is defined as a high response during the induction phase (b). In Panel **b** we define resilience, i.e. the ability to recover to the initial sate after perturbation: High resilience is defined as fast and full recovery during resolution phase (c), while low resilience is defined as slow or incomplete recovery during the resolution phase (d).

Plaque accumulation and gingival responses to the absence of oral hygiene vary from person to person [1]. However, when oral hygiene practices are restored, values of plaque index and gingival index drop rapidly, indicating a reversible inflammation mechanism of the gingiva. The most frequently used longitudinal design for modelling the early phases of gingival inflammation is the experimental gingivitis (EG) protocol, originally developed by Löe et al. to demonstrate that de novo accumulation of plaque was associated with the development of gingivitis [1]. Participants were asked to refrain from any form of oral hygiene to induce plaque accumulation until gingival inflammation occurred. The time frame for development of gingivitis varied from 10 to 21 days. Different protocols have been developed thereafter with the addition of an intervention or a pre-trial phase. This phase typically consists of professional prophylaxis to achieve zero inflamed gingival tissues so that effects of plaque accumulation alone could be studied. Eventually the induction phase ended with professional prophylaxis to restore gingival health. By demonstrating that removal of plaque resolved gingival inflammation, strong evidence was provided that plaque causes gingivitis [1]. A recent study showed little to no inflammation and no red fluorescent plaque in some participants during an EG trial, while other participants have shown high levels of plaque and gingival inflammation [2]. In this study, such subjects are referred to as high or low responders, respectively, to the exposure. Subjects can have a high resistance to perturbation or EG, in which few changes occur in the oral ecosystem, therefore will have little restoration after the trial. A low resistance means that the impact on the ecosystem is high (Fig. 1). Resilience is defined as the property of the oral ecosystem to recover to its initial state [3]. In the EG trial, participants can have a high (full) or low (incomplete) resilience after the experiment (Fig. 1). Factors associated with resilience of the periodontal/gingival tissue to perturbating factors could include genetics and/or lifestyle factors such as smoking, diet and psychosocial factors [4]. In the case of EG, the pre-existing inflammatory status and the condition of the oral cavity as a whole in the pre-trial phase could influence the outcome of the study, and thus determine resilience [5].

The EG trial has been adapted by many research groups resulting in a variety of protocols being adopted for each phase. The experiment is used to study a large diversity of outcomes from clinical, microbiological to immunologic outcomes, and subjects, from healthy subjects to the comparison of systemically unhealthy study participants or pregnant women. In this systematic scoping review we aimed to identify in EG studies changes in biomarkers of microbiological, immunological and biochemical origin that might indicate resistance and resilience. We searched for potential markers of resistance and resilience in the induction and resolution phase respectively. The retrieved evidence is tabulated and knowledge indications for resistance and resilience, or gaps in the literature, are presented for future research.

## Methods

### Study design

The preliminary assessment of the size and scope of available research literature regarding oral resilience to EG was conducted according to the JBI Briggs Reviewers Manual and the PRISMA statement for transparent reporting of systematic reviews and meta-analysis [6, 7] In order to ensure systematic reviewing of the literature, the PRISMA checklist and guideline was used throughout the review process.

### Electronic search

The search string was specified and limited to the use of the words ‘experimental gingivitis’ in parenthesis and run in PubMed from inception to April 11th, 2018. This specified search string would limit references to those focusing only on EG, leading to a large reduction in irrelevant references when compared with other search string options. The hits arising from the search strategy were exported to RAYYAN, a systematic review web application [8], for the screening process.

### Screening process

The search hits were screened independently by C.Z. and N.J. based on title, abstract, and full text and conflicting results were discussed to reach consensus. Data extraction was double checked by B.G.L. The selected studies for full text screening were exported to EndNote. Full text studies were retrieved using EndNote, Google, Research Gate or by contacting the corresponding author. References containing data on immunological, microbiological, and biochemical parameters were included. These data were required to provide an indication or indirect evidence on resistance and/or resilience of the oral cavity during and after the EG trial. Studies on mouth rinses, placebo control groups, animals, surgical treatments, antibiotics, probiotics, those lacking a healthy control population, cross-sectional designs, any form of intervention during the induction phase, and split mouth experimental design were excluded. The latter type studies were excluded due to risk of transfer of fluids and biomarkers from one part of the oral cavity to another. Moreover, systemic responses are likely to be lower in a split mouth study design than in full-mouth EG due to the smaller gingival surface area of inflammation; this may then affect the inflammatory profile of plasma and GCF.

### Data management

Data on study setup, parameters and study characteristics were reported in separate tables. The data were summarized narratively to provide an overview of retrieved evidence and reported resilience based on microbiological, immunological and biochemical origin. For each study we conducted a risk of bias assessment using the JBI checklist for analytical cross sectional studies. We have categorized the risk of bias scores as follows: < 4, high risk of bias; 4–5, moderate risk of bias; 6–7, low risk of bias.

## Results

**Fig. 2:**
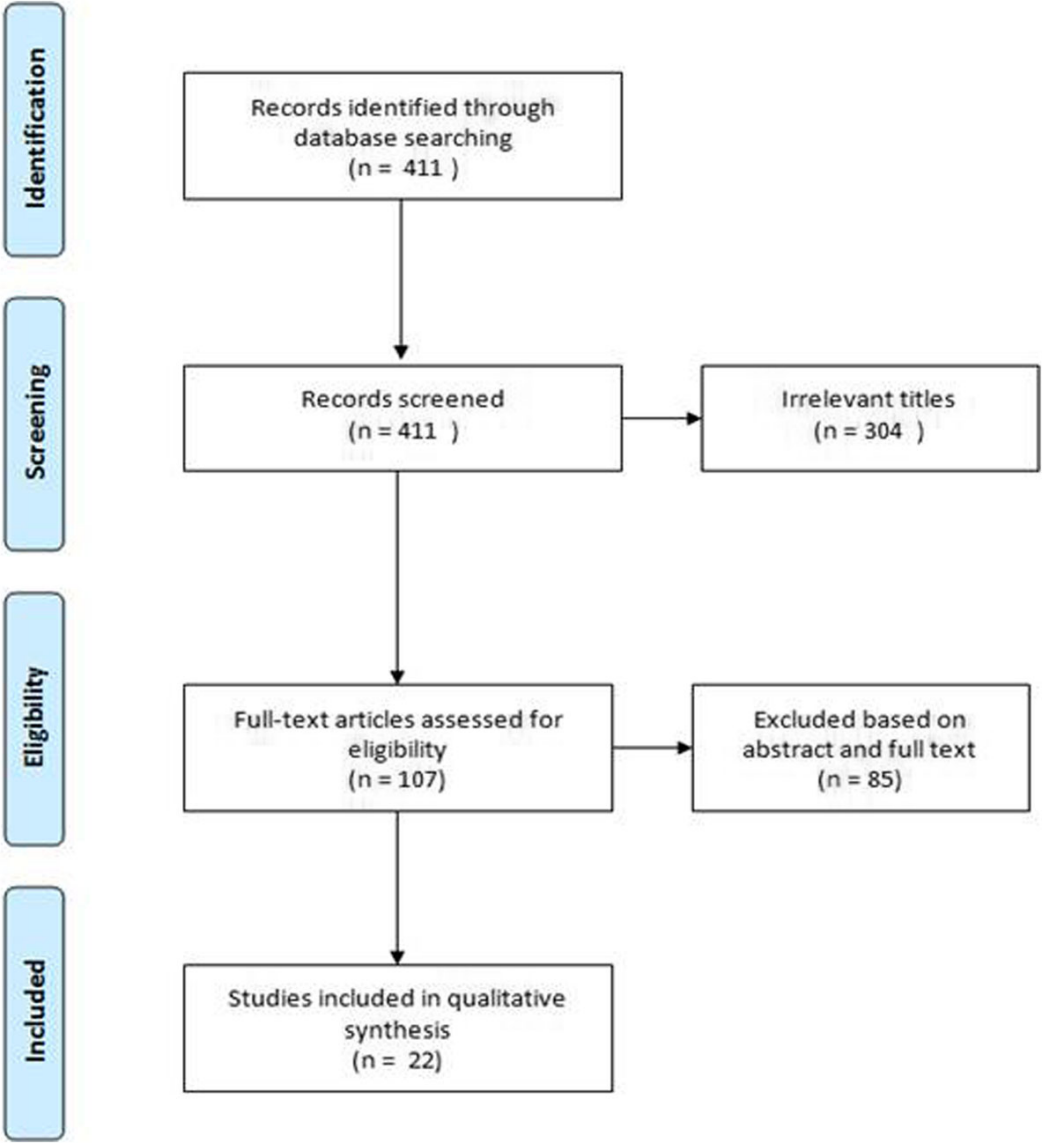
PRISMA Flowchart of the search and selection process

The search strategy yielded 411 publications, of which 104 were screened based on full text analysis. Finally, 22 unique studies were included for the current review (Fig. 2). The details of the included studies are reported in Table 1. A list of excluded studies based on full text is provided in Additional file 1. It was noted that 18 of these studies were excluded on the basis that full text was not available. These were generally in smaller subscription-only dental journals and we were unable to access the documents. In total, two studies reported microbiological findings [22, 23], eight studies studied biochemical changes [10, 12–14, 21, 24–27] and 12 analyzed immunological results [9, 11, 15–20, 28–30]. Full descriptions of all studied parameters are mapped in Additional file 2. The protocols in the included studies differed in terms of time lines, coverage of phases and comparison groups. No studies specifically set out to identify factors associated with resilience of the oral cavity to the challenge arising from abstinence of oral hygiene measures. Five studies compared smokers to non-smokers in exposure to the EG trial [18, 23–25, 29]; smoking status was always based on self-report and not biochemically assessed. A sample size of less than 20 individuals was reported in 15 studies, while the six other studies included from 21 to 156 subjects. Students were used as a study population in 12 studies, of which nine focused on dental students. Two studies examined elderly versus younger adults and one study included only children: Down syndrome and healthy. Apart from the child study (ages not reported), the youngest and oldest ages reported were 18 and 77. A total of 14 studies included a pre-trial phase, and nine studies added a resolution phase to the experiment. The study setup, per phase of the EG trial, is provided in Additional file 3.

**Table 1:** Overview of included studies: description of study characteristics and extracted data, stratifies by microbiological (A), immunological (B) and biochemical (C) outcomes

First author, year^reference^	Study population	Design set up	Parameters	Results resilience
A. Microbiological outcomes				
Lie, 1998 [6]	*N* = 25 Age: 19–27 years, mean: 22 Sex: M: 12, F:13 Groups: 1 group non-dental university students Ethnicity: NR Smoking: 11 (M:6; F:5) Average cigarettes: 15.6 a day General health: Good general health Oral health: 24 teeth, > 30% bleeding on probing, pockets < 5 mm.	Pre-trial phase: Length: NR Treatment: OHI, PP Induction phase: Length: 14 days Treatment: PP at the end Resolution phase: N/A Samples: supragingival plaque, approximal plaque, subgingival plaque, tongue scrape	- Total CFU - *P. micros* *- Streptococcus* spp. *- A. actinomycetemcomitans* *- C. rectus* *- F. nucleatum* *- P. intermedia* *- Actinomyces* spp.	Supragingival plaque No difference found in groups and over time in total CFU, *P. micros, Streptococcus* spp. *Actinomyces* spp. and *F. nucleatum, P. intermedia* increased from day 0 to day 14 in non-smokers group. *C. rectus* increased in both groups. Subgingival plaque No difference was found over time and between *P. micros, Streptococcus* spp.*, A. actinomycetemcomitans, C. rectus, F. nucleatum* groups in abundance. Total CFU in smokers was at day 14 higher compared to baseline. *Actinomyces* spp. and *P. intermedia* increased significantly over time in both groups. Approximal plaque: Total CFU, *A. actinomycetemcomitans, Actinomyces* spp., *C. rectus (*in smokers) and *F. nucelatum* (in non-smokers), *P. micros, streptococci* spp. did not change over time. *Actinomyces* spp. and *C. rectus* increased over time in non-smokers group and *F. nucleatum* in smokers group.
Salvi, 2005 [7]	*N* = 22 Age: mean 24.7 (2.2 SD) years Sex: M:7, F:15 Groups: smokers (*N* = 11) and control group of non-smokers (N = 11) all students. Ethnicity: NR Smoking: 11, > 5 years of smoking General health: No relevant medical conditions, general good health Oral health: > 24 permanent teeth, pockets < 4 mm	Pre-trial phase: Length: 21 days Treatment: PP and OHI Induction phase: Length: 21 days Treatment: none Resolution phase: Length: 14 days Treatment: OHI, PP Sample: subgingival plaque	- *P. gingivalis* *- T. denticola* *- T. forsythia*	Bacteria in smokers and non-smokers did not differ over time. At day 35 smokers had fewer bacteria compared to day 21.
B. Immunological outcomes				
Belstrom, 2017 [9]	*N* = 29 Age: 22–29 years Sex: M: 5, F:24 Groups: dental students Ethnicity: NR Smoking: non-smokers General health: Systemically healthy Oral health: No active caries, no gingivitis or periodontitis, no hyposalivation or medication use.	Pre-trial phase: Length: none Treatment: NA Induction phase: Length: 10 days Treatment: none Resolution phase: Length: 14 days Treatment: none Samples: stimulated saliva	- IL-1β - IL-8 - MCP-1 - VEGF	**IL-1β, IL-8, MCP-1,** and **VEGF** levels all reduced during induction phase compared to baseline levels. And **IL-8** remained low after resolution phase.
Brecx, 1988 [10]	*N* = 8 Age: 22–26 years Sex: NR Groups: dental students Ethnicity: NR Smoking: NR General health: NR Oral health: NR	Pre-trial phase: Length: 14 days Treatment: PP Induction phase: Length: 21 days Treatment: none Resolution phase: Length: 8 days Samples: gingival biopsy	- Lymphocytes - Macrophages - PMNs - Plasma cells	Levels of lymphocytes, plasma cells and macrophages did not change at day 8 of the resolution phase compared to end of EG, except PMNs: they increased significantly at day 8 (18.1% at end of EG, 21.9% at day 8 of resolution
Giannopoulou, 2003 [11]	*N* = 22 Age: 19–33 years, mean 26 Sex: M: 14, F: 8 Groups: dental students, 2 groups non-smokers and smokers Ethnicity: NR Smoking: *N* = 10 General health: Systemically healthy Oral health: Smokers > 10 cigarettes a day, > 24 permanent teeth, GI < 1	Pre-trial phase: Length: 12 days Treatment: PP, OHI Induction phase: Length: 10 days Treatment: PP, OHI Resolution phase: N/A Samples: GCF	- IL-1β - IL-4 - IL-8	**IL-1β** increased significantly in the induction phase in both smokers and non-smokers. **IL-4** at baseline is significantly lower in smokers compared to non-smokers and does not change over time. In non-smokers **IL-4** decreases significantly over time and stays higher compared to smokers. **IL-8** levels at baseline is significantly higher compared to non-smokers, this applies also at day 10. In both smokers as non-smokers the levels of **IL-8** increase significantly.
Heasman, 1992 [12]	*N* = 7 Age: 18–21 year Sex: NR Groups: NR Ethnicity: NR Smoking: NR General health: NR Oral health: > 20 permanent teeth, < 3 mm pocket depth, no bone loss	Pre-trial phase: Length: 7 days Treatment: PP Induction phase: Length: 28 days Treatment: PP Resolution phase: N/A Samples: GCF	- IL-1B - LTB4 - PGE2 - TxB2	**IL-1B** increased over time during the induction phase compared to baseline. **LTB4** levels differed significantly only in 7 and 28 days, in between the levels stay the same as baseline values. **PGE2** and **TxB2** values only changed compared to baseline after 28 days, before that the levels stay the same as at baseline.
Kinane, 1992 [13]	*N* = 12 Age: 20–21 years Sex: M: 8, F: 4 Groups: dental students Ethnicity: NR Smoking: NR General health: Unremarkable medical history Oral health: > 26 permanent teeth, healthy gingiva, < 4 mm pocket depth, no attachment loss	Pre-trial phase: Length: 8 weeks Treatment: monitoring oral health Induction phase: Length: 21 days Treatment: PP Resolution phase: Length: 14 days Treatment: none Samples: GCF	- IL-1	**IL-1** levels strongly increase from baseline to day 4 and stay at the same levels until day 14. The **IL-1** levels return back to baseline levels at the end of the induction phase.
Leishman, 2013 [14]	*N* = 8 Age: 25.5 (7.1 SD) years Sex: M: 0, F: 8 Groups: university students Ethnicity: NR Smoking: none General health: Systemically healthy, non-pregnant and no use of antibiotics Oral health: No history of periodontal disease	Pre-trial phase: N/A Induction phase: Length: 21 days Treatment: PP and Oral B toothbrush Resolution phase: Length: 14 days Treatment: none Samples: GCF, saliva, blood plasma	- IL-1β - IL-2 - IL-4 - IL-5 - IL-6 - IL-10 - IFN-γ - sICAM-1 - TNF-α	GCF sample: **IL-1β** significantly decreases after 35 days. **IL-2** increases from baseline to day 21 and decreases 10 fold at 35 days. **TNF-α** decreased in resolution phase and **sICAM-1** was significantly higher at day 35 compared to day 14. **IL-4, IL-5, IL-10, IL-12 and IF-γ values** stayed the same over time. Saliva sample: Only **IL-1β** significantly decreased at day 35 compared to day 21. Other parameters did not change significantly. Plasma sample: Only **sICAM-1** values changed significantly over time and increased from baseline to day 35. However the values between day 21 and 35 were not significantly different.
Lie, 2002 [15]	*N* = 25 Age: 19–27 mean 22 year Sex: M: 12, F:13 Groups: 1, non-dental university students Ethnicity: NR Smoking: *N* = 11 General health: Good general health Oral health: > 24 permanent teeth, < 4 mm pocket depth, no attachment loss	Pre-trial phase: Length: NR Treatment: PP Induction phase: Length: 14 days Treatment: PP Resolution phase: N/A Samples: stimulated saliva	- Total parotid salivary IgA	No change in **salivary-IgA** over time or difference between groups.
Norman, 1979 [16]	*N* = 10 Age: NR Sex: Male Groups: dental students Ethnicity: NR Smoking: none General health: Healthy Oral health: No periodontitis	Pre-trial phase: Length: NR Treatment: PP Induction phase: Length: 21 days Treatment: PP Resolution phase: N/A Samples: Blood and GCF	- IgA - IgM - IgG	**IgG, IgM, IgA,** showed no significant changes during the induction phase.
Reuland-Bosma, 1987 [17]	*N* = 16 Age: NR Sex: NR Groups: 2, 1 down syndrome children, 1 healthy control children. Ethnicity: NR Smoking: none General health: NR Oral health: NR	Pre-trial phase: N/A Induction phase: Length: 21 days Treatment: none Resolution phase: N/A Samples: gingival biopsies	- Leukocytes - PMNs	Number of **PMNs** and **leukocytes** did not change over time in the induction phase compared to baseline.
Seemann, 2004 [18]	*N* = 14 Age: 22–37, mean 27 years Sex: M:14, F: 0 Groups: 1 Ethnicity: NR Smoking: none General health: Clinically healthy Oral health: > 24 teeth, pockets depth < 4 mm, no active caries, good general health	Pre-trial phase: Length: 2 days Treatment: none Induction phase: Length: 12 days Treatment: PP Resolution phase: N/A Samples: (un)stimulated saliva	- Parotid IgA concentration - Parotid IgA secretion - SM/SL IgA concentration - SM/SL IgA secretion	**Parotid IgA concentration,** stayed the same during the trial. **Parotid IgA secretion** in stimulated saliva increased significantly from baseline to end of induction phase. **SM/SL IgA concentration and secretion** remained stable during the experiment.
Tsalikis, 2010 [19]	*N* = 10 Age: 20–22 (*N* = 5) and 61–65 years (*N* = 5) Sex: M:6, F: 4 Groups: 2, young adults and old adults Ethnicity: Caucasians Smoking: none General health: Clinically healthy Oral health: healthy periodontum, no bone loss, > 20 permanent teeth, attachment loss < 3 mm, bleeding on probing < 10%, plaque index < 20%	Pre-trial phase: N/A Induction phase: Length: 21 days Treatment: none Resolution phase: Length: 7 days Treatment: PP Samples: GCF	- IL-6 - IL-8 - TNF- α	**Young group: TNF- α** did not change over time. **IL-6** and **IL-8** increased from baseline to induction and to resolution phase. **Old group: TNF- α** increased from baseline to end of induction phase, and **IL-6** from end induction phase to end of resolution phase.
Wahaidi, 2009 [20]	*N* = 156 Age: 18–31 years Sex: NR Groups: non smoking Ethnicity: NR Smoking: none General health: healthy Oral health: No periodontitis, < 4 mm probing depth, no caries	Pre-trial phase: Length: 21 days Treatment: OHI Induction phase: Length: 21 days Treatment: PP, OHI Resolution phase: Length: 21 days Treatment: none Samples: peripheral blood	- White blood cells - PMNs	No significant change in either parameter over time.
Zhou, 2012 [21]	*N* = 11 Age: years Sex: M: 11, F: 0 Groups: 1 group non-smoking dental students Ethnicity: NR Smoking: none General health: Unremarkable medical history Oral health: No periodontal disease, > 24 permanent teeth, healthy gingiva, good oral hygiene	Pre-trial phase: N/A Induction phase: Length: 21 days Treatment: none Resolution phase: Length: 7 days Treatment: none Samples: unstimulated whole saliva	- IL-6 - IL-1β	**IL-6** showed increase at day 21 and returned to baseline values during the resolution phase. **IL-1 β** was significantly higher at day 14 and 21 compared to baseline, and returns to baseline values in resolution phase.
C. Biochemical outcomes				
Aboodi, 2015 [8]	*N* = 5 Age: 20–36 year Sex: M:2, F:3 Groups: NR Ethnicity: NR Smoking: none General health: Systemically healthy Oral health: Periodontally healthy	Pre-trial phase: Length: 7 days Treatment: PP, OHI Induction phase: Length: 21 days Treatment: none Resolution phase: Length: 14 days Treatment: OHI, PP Sample: whole saliva	Proteome (fold change): - SPARC-like protein 1 (2.10) - Calmodulin-like protein 3 (2.11) - Mucin-6 (2.13) - Cystatin-S (2.14) - LOC349136 (2.18) - Tetratricopeptide repeat protein 28 (2.20) - Androgen receptor (2.21) - Serine 13 (2.21) - Carbonic anhydrase 6 (2.23) - Kinesin-like protein KIF3B (2.23) adenocarcinoma factor (2.25) - Thioredoxin (2.29) - cDNA FLJ38275 (2.31) - Apolipoprotein A-1 (2.32) - Pancreatic - Albumin (2.33) - Cystatin-SN (2.33) - OLIG2 (2.47) - Vitamine D-binding protein (2.78) - IG kappa chain C region (2.87) - G-protein r98 (3.41) - Lactoferrin (a.k.a. lactotransferrin) (3.50) - Beta-globin (3.75) - Anenxin A1 (4.15) - KIAA1539 (4.72) - Collagen alpha-1 (7.35)	All reported proteins changed over time and have a fold change > 2.
Adonogianaki, 1994 [22]	*N* = 6 Age: 22–23 year Sex: M:5, F:1 Groups: dental students Ethnicity: NR Smoking: NR General health: NR Oral health: < 3 mm pocket depth, no periodontal attachment loss	Pre-trial phase: Length: 10 days Treatment: PP, OHI Induction phase: Length: 21 days Treatment: none Resolution phase: Length: 14 days Treatment: none Samples: GCF	- Alpha2-macroglobulin - Alpha1-antitrypsin - Transferrin - Lactoferrin	**Alpha1-antitrypsin, Alpha2-macroglobulin** and **transferrin** increased during the induction phase and stayed elevated in the resolution phase. **Lactoferrin** increased also during the induction phase, but dropped again almost to baseline levels during the resolution phase.
Lie, 2001 [23]	*N* = 25 Age: 17–27 mean 22 years Sex: NR Groups: 2 groups, non-smokers and smokers Ethnicity: NR Smoking: 11 General health: Good general health Oral health: having gingivitis > 30% bleeding upon probing, < 5 mm of pockets, absence of approximal attachment loss. When treated in the pre-trial phase, patients with < 20% approximal bleeding sites entered the trial	Pre-trial phase Length: NR Treatment: OHI and PP Induction phase: Length: 14 days Treatment: none Resolution phase: N/A Samples: saliva	- Total protein (mg/ml) - Cystatin activity (units/ml) - Cystatin C (μg/ml) - Output cystatin activity (units/min) - Output cystatin C (μg/min)	Mean **protein** values remained stable over time. **Cystatin activity** significantly decreased at day 14 compared to baseline in smokers. **Cystatin C** was at day 14 significantly higher in non-smokers, and decreased during induction in smokers. **Output cystatin activity** was at day 14 higher in non-smokers. **Output cystatin C** decreased during induction in non-smokers and was significantly higher in smokers at day 14
Norman, 1979 [16]	*N* = 10 Age: NR Sex: M: 10, F:0 Groups: dental students Ethnicity: NR Smoking: NR General health: NR Oral health: periodontally healthy	Pre-trial phase: Length: NR Treatment: PP Induction phase: Length: 21 days Treatment: PP Resolution phase: N/A Samples: Blood and GCF	- Alpha-antitrypsin - C4 - C3 - CH50 - Factor B - Transferrin	**C3, Factor B, Alpha-antitrypsin** and **transferrin** showed no significant changes during the induction phase. **CH50 and C4** increased significantly at day 21 compared to baseline values.
Ozdemir, 2009 [24]	*N* = 12 Age: 19–21 years Sex: M: 12; F:0 Groups: dental students Ethnicity: NR Smoking: none General health: NR Oral Health: > 24 permanent teeth, systemic healthy, < 4 mm pocket depth, no attachment loss	Pre-trial phase: Length: 14 days Treatment: OHI, PP Induction phase: Length: 14 days Treatment: OHI, PP Resolution phase: Length: 21 days Treatment: none Samples: GCF, peripheral blood	- Lactoferrin	**Lactoferrin** in GCF and blood increased during the induction phase and returned to baseline in the resolution phase.
Que, 2004 [25]	*N* = 15 Age: 18–30 years Sex: M: 6, F: 9 Groups: NR Ethnicity: NR Smoking: none General health: Systemically healthy no NSAIDs use. Oral health: < 5 mm pocket depth, no attachment loss	Pre-trial phase: Length: 11 days Treatment: PP and OHI Induction phase: Length: 10 days Treatment: PP Resolution phase: N/A Samples: GCF	- Total protein (ng/site) - Calprotectin (MRP8/14) (ng/site) - subunit MRP 8 (ng/site) - subunit MRP 14 (ng/site)	**Total protein, calprotectin, MRP 8** and **14** did not change from baseline to day 10. However 1 day after cleaning this did increase significantly.
Siegel, 2007 [24]	*N* = 14 Age: 18–30 and 46–77 years Sex: M: 9, F: 5 Groups: 2 groups, 18–30 year old subject and 47–77 year old. Ethnicity: NR Smoking: none General health: No systemic diseases Oral health: < 3 mm pocket depth, no bleeding on probing, non-susceptible for periodontitis, > 20 permanent teeth, no systemic disease	Pre-trial phase: Length: 21 days Treatment: OHI and PP Induction phase: Length: 14 days Treatment: PP Resolution phase: N/A Samples: gingival biopsy	- Cyclooxygenase − 1 - Cyclooxygenase − 2- Cyclooxygenase − 3 - Microsomal prostaglandin e synthese-1	**Cyclooxygenase − 1** expression in Langerhans’cells increased in both study groups but was not significant. **Cyclooxygenase − 2** expression in basal epithelial cells were significantly lower in the older age group. **Cyclooxygenase − 3** was not detected in tissue samples. **Microsomal prostaglandin E synthese-1** was detected in epithelial, endothelial and fibroblast-like connective tissue cells, however no significant difference was found between ages.
Uitto, 1996 [26]	*N* = 12 Age: NR Sex: NR Groups: 2 groups, student hygienists and healthy adults Ethnicity: NR Smoking: NR General health: NR Oral health: healthy periodontium	Pre-trial phase: N/A Induction phase: Length: 10 days Treatment: none Resolution phase: N/A Samples: saliva samples; water rinses	- Elastase	No significant changes in **salivary elastase** levels during the experiment.
Zhou, 2012 [21]	*N* = 11 Age: 21–22 years Sex: M: 11, F: 0 Groups: 1 group non-smoking dental students Ethnicity: NR Smoking: none General health: Unremarkable medical history Oral health: No periodontal disease, > 24 permanent teeth, healthy gingiva, good oral hygiene	Pre-trial phase: N/A Induction phase: Length: 21 days Treatment: none Resolution phase: Length: 7 days Treatment: none Samples: unstimulated whole saliva	- Calprotectin - Elastase activity	**Calprotectin** showed only change at day 21,and returned to baseline levels. Elastase activity was higher in the induction phase compared to resolution phase.

*Abbreviations*: *M* male, *F* female, *NR* not reported, *OHI* oral hygiene instruction, *PP* professional prophylaxis, *N/A* not applicable, *OH* oral hygiene, *GCF* gingival crevicular fluid, *SD* standard deviation, *GI* gingival index, *IL-* interleukin, *PMN* polymorphonuclear neutrophils, *TNF* tumour necrosis factor, *mm* millimetres, *VEGF* vascular endothelial growth factor

### Risk of bias assessment

Ten studies had a moderate risk of bias (scores 4–5) and 11 studies had a low risk of bias (scores 6–7). None of the studies had taken confounding factors into account in the final analysis, therefore no score ‘7’ was reached. The complete risk of bias table is provided in Additional file 4.

### Microbiological markers

Two studies investigated microbiological changes during the EG trial and compared the outcomes in smokers and non-smokers [22, 23]. Both studies were almost equal in size, and both had professional prophylaxis in the pre-trial phase.

The study of Salvi et al. [23] using DNA-DNA checkerboard hybridization techniques on subgingival plaque samples, showed no differences in total DNA probe counts during the induction phase (day 0 compared with day 21) and after resolution (day 35 compared with day 21). Species were grouped on the basis of the colour-coded complexes identified by Socransky et al. [31] in a checkerboard hybridization study of bacteria associated with periodontal disease. Purple complex species (*Veillonella parvula* and *Actinomyces odontolyticus*) were significantly decreased in non-smokers at day 21 compared with baseline. *Actinomyces* sp. (blue complex) and the yellow complex, containing oral streptococci *Streptococcus sanguinis*, *S. oralis*, *S. mitis*, *S. gordonii* and *S. intermedius*, increased in non-smokers during resolution. By contrast, red complex species (*Porphyromonas gingivalis, Treponema denticola,* and *Tannerella. forsythia*) that are most strongly associated with periodontitis were significantly elevated in smokers in subgingival plaque by 21 days of EG, and significantly reduced over the resolution phase, from day 21 to day 35, in both smokers and non-smokers [23].

The study of Lie et al. [22] employed traditional microbiological culture and identification to investigate nine different microbial taxa in three different types of dental plaque: supragingival, subgingival or approximal, including swabs of the tongue and tonsils. In supragingival dental plaque samples, the total CFU increased significantly from baseline to the end of the EG phase (day 14) in non-smokers from 1.1 × 10^6^ CFU to 3.7 × 10^6^ CFU. By contrast, there was not a significant difference over this phase in smokers. *Aggregatibacter* (formerly *Actinobacillus*) *actinomycetemcomitans*, *Parvimonas micra* (formerly *Peptostreptococcus micros*) and *Streptococcus* spp. did not differ between groups or over time in the induction phase. Similarly, there was no significant difference over time in levels of *A. actinomycetemcomitans, Campylobacter rectus, Fusobacterium nucleatum, Streptococcus* spp., and *P. micra* in subgingival plaque, and total CFU, *A. actinomycetemcomitans, Streptococcus* spp., and *P. micros* in approximal plaque [22]. The mean total CFU of *Campylobacter rectus* and *F. nucleatum* increased during EG in smokers and these species, together with *Actinomyces* spp. and *P. intermedia*, increased in non-smokers. Differences varied from a 3.4-fold increase in *Actinomyces* spp. in non-smokers between day 0 and 14 to a 29-fold increase in *P. intermedia* in non-smokers over the same timeframe. There was a significant increase in total CFU in subgingival dental plaque in smokers, but not in non-smokers, during EG, from 6.3 × 10^5^ CFU to 3.4 × 10^6^ CFU. *Actinomyces* spp. and *P. intermedia* were increased in both smokers and non-smokers. In approximal dental plaque, *Actinomyces* spp. and *C. rectus* were increased by the end of EG in non-smokers, *F. nucleatum* was increased in smokers and *P. intermedia* was increased in both smokers and non-smokers. There were no significant changes in microbial counts between the start and end of EG in samples from the tongue or tonsils [22].

Overall, these two studies [22, 23] demonstrate that the microbiological composition of both supragingival and subgingival dental plaque changes over the course of EG and during resolution, and the changes appear to be affected by smoking status. However, the study of Salvi et al. [23] did not analyse changes in individual species, and it is therefore impossible to identify consistent markers of responsiveness or resilience on the basis of these two studies alone.

### Immunological markers

#### Biopsies and systemic findings

Three studies investigated leukocyte changes in the EG trial [14, 20, 28]. They had a 21-day induction phase and the levels of leukocytes, both in gingival biopsies and peripheral blood, did not change in the induction phase compared to baseline levels. Only in one study (*N* = 8) polymorphonuclear neutrophils (PMNs) in gingival biopsies were found to increase significantly from 18.1% at the end of the EG trial (i.e. day 0 of resolution phase) to 21.9% at day 8 of the resolution phase [14]. This observation is counter intuitive, however the difference between 18.1 and 21.9% may not be clinically significant; fibroblasts, lymphocytes, plasma cells and macrophages did not show significant changes in the resolution phase.

As a result from inflammatory reactions, sICAM is known to increase in plasma. In one study, sICAM-1 in plasma, increased significantly 2.6-fold in the induction phase and remained elevated during the 2 weeks of resolution phase [14]. The results for sICAM-1 in EG and its resolution indicate a low resistance and low resilience to the occurring gingival inflammation. Here we found another example where a biomarker remained longer present in plasma while the gingiva has returned to its healthy state. Interestingly, for the cytokine interferon-γ there were no significant changes during the induction and resolution phases [14], suggesting no major involvement of this immune mediator in EG.

One study looked for immunoglobulins in serum. The values of IgG, IgM and IgA of the EG subjects compared to subjects who maintained normal oral hygiene, remained stable during the induction period [19]. No resolution phase was included.

#### Saliva

In the study of Zhou et al. [13], expression of interleukin (IL)-1β in unstimulated whole saliva was significantly higher during the induction phase and returned to baseline levels at resolution suggesting that gingival health improved at the end of the resolution phase to a (more) healthy state and shows normal resilience and inflammatory response to the challenge. In one small study of Belstrøm et al. [30], IL-β levels in stimulated saliva were decreased after 10 days of induction phase compared to baseline levels. Mean IL-6 in whole saliva increased significantly from 6.70 pg/mL at baseline to 7.86 pg/mL at day 21 (end of induction). The values decreased to 7.05 pg/mL in the resolution phase, which was not significantly different compared to day 0. Further, IL-8 levels in stimulated saliva were reduced after 4 days of induction and remained low up to resolution phase [30]. Notably, IL-2, IL-4, IL-5 and IL-10 in saliva did not significantly change over time [12].

Immunoglobulins (Ig) were examined in three studies [11, 18, 19]. IgA [19] secretion in stimulated parotid saliva increased significantly from 20 μg/mL to 40 μg/mL in the induction phase in a healthy study group. However, the IgA secretion and concentration in resting parotid saliva, submandibular/sublingual saliva (both resting and stimulated) remained stable in this stage [18]. No data were reported on the resolution phase [11].

#### Gingival crevicular fluid

Various IL were studied in six studies [13, 15–17, 19, 28, 29]. In two of these studies [13, 19], no changes in IL-4, IL-5, IL-6, IL-8, IL-10 and IL-12 in gingival crevicular fluid (GCF) were found during the induction phase. In the study of Heasman et al. [15], IL-1β in GCF from 20 pooled sites/subject showed almost 8-fold increase from 16.5 ng/mL at baseline to 131 ng/ml at week 1, and thereafter remained approximately at that level until the end the experiment (28 days). A similar pattern was reported by Leishman et al. [17]; interestingly they included a resolution phase in their study and found that IL-1β in GCF showed a decrease, beyond the levels of baseline (52.91 pg/mL) to 9.64 pg/mL at the end of resolution phase. In smokers participating in an EG trial, the level of IL-1β in GCF, increased from baseline to day 10 of the induction phase, from 6.2 pg/20s to 13.6 pg/20s, while in non-smokers the respective values were 3.99 pg/20s to 9.8 pg/20s [19].

Another interleukin, IL-2 in GCF, increased from 7.81 pg/mL at baseline to 11.82 pg/mL at the end of the induction [13]. However, IL-2 levels dropped to below baseline measurements of 0.59 pg/mL [13], suggesting a return to normal non-inflamed gingiva and that gingival tissues at baseline were somewhat inflamed to begin with.

The output rate of IL-4 was found in GCF to decrease after 10 days of induction phase in non-smokers from 10.2 pg/20s to 5.5 pg/20s, and remained stable in smokers: 4.4 pg/20s to 4.1 pg/20s [19]. The response of IL-4 is inconsistent with the data of Leishman et al. [17]. In this study, the induction phase consisted of 21 days in which the level of IL-4 in GCF did not significantly change over time. However, it did drop to 0 after 14 days of resolution indicating resilience to the trial by marker IL-4.

For IL-8 the output rate in GCF increased significantly during the induction phase in smokers and non-smokers (21.1 pg/20s to 56.1 pg/20s; 15.1 pg/20s to 36.5 pg/20s respectively), with a significant higher increase in smokers [29]. This might indicate that smokers have a higher host response than non-smokers to an inflammatory challenge.

The study conducted by Tsalikis et al. [28] compared TNF-α levels in GCF in old and young participants (61–65 and 20–22 years). The values at baseline did not differ between age groups. At day 21 of the induction phase, and at day 7 of the resolution phase, the values were significantly higher in the elderly participants, while the younger participants did not show any changes over the total experiment. This suggests that older age is associated with a lower resistance to EG challenge than younger age. The latter results are somewhat different from another study: in the study conducted by Leishman et al. [17] with a study population of a mean age of 25 years, the TNF-α values in GCF increased during induction of a 3 week period (0.98 pg/mL to 1.75 pg/mL) and decreased to 0.11 pg/mL in the resolution phase, i.e. indicating a high resilience. However the contradictory results for TNF-α in GCF between the two studies [17, 28], make it difficult to draw a conclusion on the overall response to EG.

Only one study investigated the lipid immune mediators, PGE-2 and TxB2 in GCF samples. The result showed that these mediators were not elevated during the first 3 weeks. However, the values increased significantly at the 4th week of the induction phase, suggesting that resistance to EG may be weakened when the EG was extended beyond the traditional 3 week period [15].

### Biochemical markers

#### Saliva

A full proteome analysis of whole saliva was conducted in healthy adults [27]. Several proteins, listed in Table 1, were found to show at least a two-fold increase in concentration during the induction phase. Of these, 10 proteins were identified having roles in protection of cells and tissues: vitamin D-binding protein, thioredoxin, lactoferrin (a.k.a. lactotransferrin), a G protein coupled receptor, cystatin-SN, cystatin-S, collagen alpha 1(XXVII) chain, beta-globin, annexin A1, and ALB protein. No data were reported on proteome change in the resolution phase.

Salivary cystatin activity, output and total protein were investigated in one EG study consisting of smokers and non-smokers [24]. The mean protein concentration in both groups did not significantly change over time. In non-smokers, cystatin activity, output cystatin activity, cystatin C and output cystatin C remained stable during the induction phase. However, in smokers, a significant decrease of cystatin activity from 36.2 to 28.8 (units/mL) and decrease of output cystatin C (0.37 to 0.22 μg/min) was observed at day 14 compared to baseline. When changes at day 14 were compared between groups, non-smokers showed to have a higher level of cystatin activity (49 vs 28.8 units/ml), cystatin C (0.63 vs 0.44 μg/ml), output cystatin activity (31.6 vs 17.5 units/min), and output cystatin C (0.46 vs 0.22 μg/min). We noted from this one study on salivary cystatin parameters a tendency for these biomarkers in non-smokers to remain stable during the induction phase of EG, indicating that perhaps these salivary factors do not play a major role in resistance to EG. On the other hand, these parameters go down in smokers, which may indicate that smokers have less salivary protection capacity in an EG trial.

From the EG trial by Uitto et al. [12], concentrations of salivary elastase levels in oral rinse samples were measured at day 10 and day 15 of the induction phase, and no significant differences were reported over time. In another study, elastase activity in saliva samples increased significantly at day 2 of the induction phase and remained elevated until day 14, while it decreased again in the resolution phase to baseline levels [13]. These results indicate that elastase activity is a useful biomarker for resistance and resilience of EG in periodontally healthy individuals.

#### Gingival crevicular fluid

An EG study conducted in 12 healthy male dental students, measured levels of lactoferrin in GCF and found significant increase from day 0 to the end of induction phase (14 days) of 58.8 and 163.2 ng/μl, respectively [26]. This was consistent with the results found by Adonogianaki et al. [25], who also conducted their study with GCF from healthy dental students (*N* = 6). In the resolution phase, the lactoferrin levels returned to baseline values. Lactoferrin in GCF might be a good biomarker to indicate resistance (increase of antibacterial activity) and resilience in EG.

In one EG study (*N* = 10), transferrin levels showed no significant changes in blood and GCF during induction phase [18]. This is inconsistent with the study of Adonogianaki et al. [25], in which transferrin levels seemed to increase significantly over time, but did not return back to baseline levels after resolution.

Calprotectin is an acute phase reactant to an inflammation associated with tissue destruction and neutrophil action. Changes in calprotectin values reflect the degree of gingival inflammation. The values showed to increase in value over three-week EG period to 5 μg/ml indication an inflammation [13]. After 1 week of resolution phase, the inflammatory biomarker decreased to 1.7 μg/ml which was lower than baseline value of 2.4 μg/ml. The latter indicates that the oral cavity has a natural response to inflammation and restores to improved state than baseline. These findings were similar in a study in which the calprotectin levels were measured in GCF during induction phase [21]. However, the latter study included professional prophylaxis at the end of the induction, where the levels increased significantly 1 day after the intervention. This protein might not be a marker for the level of gingival inflammation during EG-trial, to indicate either resilience or resistance.

#### Biopsies and systemic findings

Lactoferrin in blood samples increased significantly from 112 ng/μl at day 0 to 200 ng/μl after 2 weeks of induction phase and reduced to baseline values in the resolution phase [26]. Similar to lactoferrin in GCF, sample values from blood might also be a good sample location to identify resilience to EG.

Norman et al. [19] studied the changes of CH50, and complement component C4 in serum for 21 day induction phase, and no resolution. The quantity increased significantly at the end of induction compared to baseline. The complement component molecule C3, Factor 8, alpha-antitrypsin, microsomal prostaglandin-E synthase-1, and cyclooxygenase-2, did not change in value during the induction phase [16, 21, 24].

One study reported monocyte chemoattractant protein-1 (MCP-1) and vascular endothelial growth factor (VEGF) in dental students undergoing the EG-trial [30]. There was a reduction of both markers during induction phase. The latter study did not include a resolution phase. No clear statements can be made on the markers as indicators of resistance or resilience during EG trial.

## Discussion

This systematic scoping review aimed to identify biological changes in the oral cavity during EG studies that might indicate resistance and resilience. EG was chosen because it is a well-established longitudinal model that has been used by many researchers. The search resulted in studies with a large variety of modifications to the original EG study [1]. In order to reduce the level of heterogeneity, studies with split mouth experiments were excluded as outlined in the Methods section (risk of transfer of fluids/biomarkers, systemic responses). Despite this, the results of the full mouth experiments were difficult to compare with each other. The set-up of the EG-phases and duration differed between studies. None of the studies aimed to study resistance and/or resilience to EG. Nevertheless the included studies gave some insights in changes during the EG challenge. The magnitude of changes might indicate either high or low resistance and/or resilience to the challenge. Only a small number of studies investigated ‘recovery’ in the resolution phase, therefore succeeded to identify markers which could be translated to resilience. The findings reported in this review regarding resistance and resilience are obviously dependent on the trial duration. For example, the study of Heasman et al. [15] showed that extending the EG trial to 4 weeks yields different results than trials of 2 weeks. This might explain why in studies with short induction and resolution phases, no clear results were found.

Only two of the included studies investigated the microbiological changes in an EG-trial [22, 23]. Using microbiological culture, the most consistent change during EG was an increase in *P. intermedia*, which occurred in 5/6 plaque samples tested (supragingival plaque from non-smokers, and subgingival and approximal plaque from smokers and non-smokers) [24]. *P. intermedia* is a member of Socransky’s orange complex [31]. When this group was considered, it was not found to change during induction or the resolution phase [23]. *Actinomyces* spp. increased consistently during the induction of gingivitis in non-smokers, and in the subgingival dental plaque of smokers [22]. *Actinomyces* spp. may provide a useful indicator of resistance, dependent on the sampling site and smoking status. However, this suggestion needs to be interpreted with care since it was found only in one study with a limited number of smokers (*n* = 11) and non-smokers (*n* = 14). Moreover, we acknowledge that when reviewing/reporting on studies involving smokers biochemical determination of smoking status would be ideal rather than the commonly applied self-reporting method. With regard to assessing resilience on the basis of commensal beneficial oral bacterial species, there is a lack of evidence from full mouth EG studies examining changes in the microbiome. More detailed studies are urgently needed using modern next generation sequencing approaches to study changes in the full microbiome of gingival sites and other sites in the oral cavity and saliva. So far, this has only been done in a study of split mouth design and focused on the induction phase alone [32].

Several immunological markers have been studied during the course of EG and the subsequent resolution phase. In short, it was virtually impossible to retrieve a robust pattern of changes for one or several of these markers across multiple studies: the markers were rarely measured in the same EG design, validated in sufficient number of studies, in a variety of media (biopsy, GCF, stimulated whole saliva, unstimulated whole saliva, parotid saliva, submandibular saliva) and the reported units of interest varied for the same marker making overall conclusions difficult. One study [13] showed some increase in the number PMNs in gingival biopsies in the induction phase of EG, but the increase was not clinically relevant.

Four studies [12, 13, 15, 28] support that IL-1β appears as a normal inflammatory reaction to the challenge, with significantly higher values in smokers. The same parameter is a marker for resilience since the values drop to below baseline levels. The same applies to IL-6 [12]. Also, IgA levels may increase in the resolution phase, but this was dependent on the analysis of either stimulated or unstimulated saliva. It is not clear how closely the amount of plaque (microbial load) is linked to IgA secretion. In summary, we can conclude that the usual pro-inflammatory markers may increase during the induction phase of EG as a resistance marker, but this is not consistently reported and gives no clues on true mechanistic pathways related to susceptibility to the levels of EG that develops in any given individual, let alone that we have seen patterns that could indicate a certain level of resilience to EG.

A number of biochemical markers in saliva have been studied at the level of protein concentration or enzyme activity during the course of EG. Proteomic analysis has highlighted factors associated with cell and tissue protection as targets of regulation during EG [27]. The samples for this study were stimulated whole saliva. Concentrations of the protease inhibitors cystatin-SN and cystatin-S were increased > 2-fold at day 21 compared to baseline. Cystatins have antibacterial activity against certain perio-pathogens [33, 34]. Therefore, increased cystatin activity is an indicator of resistance to perturbation. Several studies have shown that total cystatin activity is elevated in saliva of periodontitis patients compared with controls [33, 35, 36]. However, the picture is not entirely clear as other studies have shown reduced total cystatin activity and specific reductions in cystatin-SN and cystatin-S in periodontitis patients [37, 38]. Although our study excluded split mouth designs, it is important to note that cystatins-B and -S were essentially unchanged in GCF during 21 days of EG in one split mouth design study [39]. The expression of cystatins may depend to some extent on smoking status. Cystatin C levels were relatively constant during EG in non-smokers, but reduced significantly over this time in smokers [24]. Based on this finding, smoking is a factor leading to low resistance of the oral cavity, perhaps also reduce resilience. Clearly, more work is required to determine whether cystatin expression can be a useful indicator of resilience, and again, a biochemical confirmation for smoking/non-smoking would be best.

Metal ion sequestration is thought to be an important antimicrobial function in body fluids. Lactoferrin levels in GCF and in whole saliva increased during EG [25, 26]. It is possible that the local concentration of iron-binding chelators in the oral cavity is important to prevent the overgrowth of iron-requiring periodontal pathogens such as *P. gingivalis*. If so, the concentration of these proteins or the total iron sequestration capacity of saliva or GCF may correlate with resistance against the induction of gingivitis. However, it is important to note that these studies did not measure the total iron availability in saliva or GCF and it is possible that the leakage of haem iron via GCF from blood into these fluids may have led to increased metal ions despite the elevated levels of chelators. Calprotectin is another metal ion chelator, however its oral and salivary levels were not so clear as lactoferrin. One study found that calprotectin levels spiked briefly at the end of the induction phase or immediately following professional cleaning [12, 21]. It could be speculated that there are sensors in the oral cavity to change salivary output of important antibacterial peptides during EG.

Several other biochemical markers with potential for resistance and resilience to gingivitis have been identified, and some of these have been noted in more than one study. Unfortunately, none of the studies looking at biochemical markers reported the details of individual responses to the EG protocol or of the rate of recovery of markers during the resolution phase. Therefore, there is still extensive work needed to identify potential biochemical markers of responsiveness and resilience against EG.

Thus, the heterogeneity within the methods of the included studies have made it difficult to compare results or to draw firm conclusions based on consistent findings. The limitations to compare the studies between themselves were mainly in the differences in sample site, duration of the trial, set-up of the trial (before, during and after). For example, not all studies included a pre-trial phase involving professional prophylaxis to reach a non-inflammation status prior to the induction phase. This might have influenced the results and differences between the results when compared. Also, none of the studies conducted power calculations, most likely resulting in underpowered results. Thus, the high level of no significant changes found in many studies that did have a resolution phase could have been due to the low power. Furthermore, none of the studies used the terms resistance or resilience, or specifically looked for oral resistance and resilience. Resilience is a relatively new approach to oral health. Therefore, the interpretation of resistance and resilience are drawn by the current authors based on the findings and assumptions.

In light of the increasing evidence of different population groups responding differentially to EG, the results of the current systematic review have to be treated with caution until additional evidence provides a clear picture on which of the discussed outcomes are characteristic for specific populations. The majority of EG studies recruited healthy young adults; it is highly questionable whether the results on those subjects can be generalized to other age groups, differences in resistance and resilience to EG can be expected in older and younger participants. Indeed, in one study the TNF-α levels in GCF indicated lower resistance to EG in older participants, while in another study on the same biomarker, young adults showed a resilience in the resolution phase [17, 28]. Another difference between study groups was smoking. A well characterised difference is the bleeding response in an EG trial in smokers vs. non-smokers [23, 26]. We extracted from studies that smokers have a lower level of both cystatin C activity and cystatin C salivary output than non-smokers, without corresponding differences for the other parameters such as plaque index, parotid flow, salivary flow rate and total salivary protein [40].

In general we have noted from the current scoping review, that the great majority of EG studies employed a low to very low number of participants and essentially all were underpowered. It is of high importance to draw robust conclusions on the resistance and resilience of the oral cavity eco-system and the gingival tissues, that future EG studies follow the original EG-model including a resolution phase, applying power calculations to determine the appropriate population size. Also, for future studies, smoking status should be biochemically assessed to exclude misclassifications.

From our scoping review it is clear that currently we miss the real insight on resistance and resilience of gingival tissues and many other components of the oral cavity to the EG challenge. The results which we extracted are in fact ‘snapshots’ of non-fully comparable situations of EG. These have led to our biggest challenge to draft a theory on resistance and resilience to EG. In other words, the studies included do not allow us to determine a clear picture on the oral ecological resistance and resilience, as certain biomarkers show different results in most studies being also often of dissimilar design.

## Conclusions

This study has highlighted a number of potential microbiological, immunological and biochemical markers that may be useful for characterising the extent of resistance or resilience to the EG challenge. Further studies are now required to determine the levels of individual variation in these parameters over the course of EG and through resolution following the restoration of oral hygiene. Ultimately, it is important to understand individual response to plaque accumulation in order to better manage dental care and how we can interfere to create resistant and resilient oral ecosystems.

## Additional files

Additional file 1: List of excluded studies and reasons for exclusion (DOCX 53 kb)

Additional file 2: Tabulation of parameters included in the final analysis and their main functions (DOCX 45 kb)

Additional file 3: Summary of the EG trial design of included studies, stratified on the basis of healthy subjects and smokers vs non-smokers (DOCX 41 kb)

Additional file 4: Methodological quality and potential risk of bias assessment for the included studies. (DOCX 45 kb)

## Data Availability

Not applicable.

## Abbreviations

ALB: Albumin
CFU: Colony Forming Unit
CSF: Colony stimulating factor
EG: Experimental gingivitis
GCF: Gingival crevicular fluid
ICAM: Intracellular adhesion molecule
Ig: Immunoglobulins
IL: Interleukin
MCP-1: Monocyte chemoattractant protein-1
PGE-2: Prostaglandin-E2
PMN: Polymorphonuclear neutrophil
TbX2: Thromboxane 2
TNF-α: Tumor necrosis factor alpha
VEGF: Vascular endothelial growth factor
