# Supplementary material for: Resistance and resilience to experimental gingivitis: a systematic scoping review
Source: BMC Oral Health. 2019 Sep 11;19:212. doi: 10.1186/s12903-019-0889-z (PMC6737651; doi:10.1186/s12903-019-0889-z)
Supplement: Supplementary file 1 — List of excluded studies and reasons for exclusion (DOCX 53 kb) [file 12903_2019_889_MOESM1_ESM.docx]

**Additional file 1**

| Author, year | Title | Reason for exclusion |
| --- | --- | --- |
| Barker, 2015 | Response of chronic gingivitis to hygiene therapy and EG. Clinical, microbiological and metabolomic changes. | No full text available |
| Bergstorm, 1986 | The influence of cigarette smoking on the development of EG. | No full text available |
| Biesbrock, 2000 | Relationship of surface epithelium concentrations of IL-1 alpha and IL-1 beta to clinical inflammation during EG. | No full text available |
| Brecx, 1988 | Stereological observations on long-term EG in man | No full text available |
| Bostanci, 2013 | Label-free quantitative proteomics reveals differentially regulated proteins in EG. | Split mouth |
| Condacci, 1982 | Alpha 2-macroglobulin in sulci from healthy and inflamed human gingivae. | Split mouth |
| Danielsen, 1933 | Serum immunoglobulin G antibodies to *Porphyromonas gingivalis*, *Prevotella intermedia*, *Fusobacterium nucleatum* and *Streptococcus sanguis* during EG in young adults. | No full text available |
| Dommisch, 2015 | Expression of antimicrobial peptides and interleukin-8 during early stages of inflammation: an EG study. | Split mouth |
| Eberhard, 2013 | EG induces systemic inflammatory markers in young healthy individuals: a single-subject interventional study. | Split mouth |
| Fransson, 1999 | Differences in the inflammatory response in young and old human subjects during the course of EG. | Split mouth |
| Gaumer, 1976 | Indirect blastogenesis of peripheral blood leukocytes in EG. | No outcome of interest |
| Giannopoulou, 1992 | Neutrophil elastase and its inhibitors in human gingival crevicular fluid during EG. | Split mouth |
| Glenwright, 1996 | Chemiluminescent assay of alkaline phosphatase in human gingival crevicular fluid: investigations with an EG model and studies on the source of the enzyme within crevicular fluid. | Split mouth |
| Gonzales, 2001 | Concentration of interleukin-1beta and neutrophil elastase activity in gingival crevicular fluid during EG. | Split mouth |
| Grant, 2010 | Proteomic analysis of a noninvasive human model of acute inflammation and its resolution: the twenty-one day gingivitis model. | No outcome of interest |
| Henry, 1987 | Relationships of serum opsonins and complement in human EG. | No outcome of interest |
| Hoerauf, 2015 | Neutrophil extracellular trap formation in supragingival biofilms. | Split mouth |
| Holthuis, 1977 | Interepithelial lymphocytes in EG in young and elderly individuals. | No outcome of interest |
| Johnson, 1997 | EG in periodontitis-susceptible subjects. | No healthy control |
| Jonsson, 2011 | Gingival tissue transcriptomes in EG. | Split mouth |
| Kinane, 1994 | Acute-phase proteins in gingival crevicular fluid during experimentally induced gingivitis. | No full text available |
| Kinane, 1991 | Immunocytochemical characterization of cellular infiltrate, related endothelial changes and determination of GCF acute-phase proteins during human EG. | No full text available |
| Kistler, 2013 | Bacterial community development in EG. | Split mouth |
| Kowashi, 1979 | Increase of free collagenase and neutral protease activities in the gingival crevice during EG in man. | No full text available |
| Kowashi, 1980 | Sulcular polymorphonuclear leucocytes and gingival exudate during EG in man. | Split mouth |
| Kolb-Bachofen, 2007 | Comparison of EG with persistent gingivitis: differences in clinical parameters and cytokine concentrations. | No healthy control |
| Konradsson, 2007 | Dental biofilm, gingivitis and interleukin-1 adjacent to approximal sites of a bonded ceramic. | No healthy control |
| Kowolik, 2001 | Systemic neutrophil response resulting from dental plaque accumulation. | No outcome of interest |
| Kunimatsu, 1990 | Granulocyte medullasin levels in gingival crevicular fluid from chronic adult periodontitis patients and EG subjects. | No outcome of interest |
| Kunimatsu, 1995 | Identification and possible function of cathepsin G in gingival crevicular fluid from chronic adult periodontitis patients and from EG subjects. | No outcome of interest |
| Kunimatsu, 2990 | Cathepsins B, H and L activities in gingival crevicular fluid from chronic adult periodontitis patients and EG subjects. | No outcome of interest |
| Lamster, 1985 | Lactate dehydrogenase, beta-glucuronidase and arylsulfatase activity in gingival crevicular fluid associated with EG in man. | Split mouth |
| Lang, 1976 | Lymphocyte response to T-cell mitogen during EG in humans. | No outcome of interest |
| Lee, 2015 | Modulation of the host response by probiotic *Lactobacillus brevis* CD2 in EG. | No outcome of interest |
| Lehner, 1974 | Sequential cell-mediated immune responses in EG in man. | No outcome of interest |
| Li, 2014 | Evaluation of the antigingivitis effect of a chlorhexidine mouthwash with or without an antidiscoloration system compared to placebo during EG. | No full text available |
| Lie, 2001 | Occurrence of *Prevotella intermedia* and *Prevotella nigrescens* in relation to gingivitis and gingival health. | No outcome of interest |
| Lie, 2001 | Salivary cystatin activity and cystatin C in EG in non-smokers. | Double |
| Lie, 1995 | Oral microbiota in subjects with a weak or strong response in EG. | Split mouth |
| Loesche, 1987 | Bacteriology of human EG: effect of plaque and gingivitis score. | No outcome of interest |
| Matthews, 2013 | Host-bacterial interactions during induction and resolution of EG in current smokers. | Split mouth |
| Mengel, 2009 | Effect of age on gingival crevicular fluid concentrations of MIF and PGE2. | No outcome of interest |
| Michelet, 1991 | Extracellular matrix and intermediate filaments in the first stages and repair of EG in man. | No full text available |
| Moore, 1982 | Bacteriology of EG in children. | No outcome of interest |
| Moore, 1984 | Bacteriology of EG in young adult humans. | No outcome of interest |
| Moore, 1987 | Bacteriology of human gingivitis. | No full text available |
| Moughal, 1992 | Langerhans cell dynamics in human gingiva during experimentally induced inflammation. | No full text available |
| Moughal, 1992 | Endothelial cell leukocyte adhesion molecule-1 (ELAM-1) and intercellular adhesion molecule-1 (ICAM-1) expression in gingival tissue during health and experimentally-induced gingivitis. | No full text available |
| Nord, 1971 | Enzyme activities in EG in man. | No full text available |
| Nylander, 1993 | Expression of the endothelial leukocyte adhesion molecule-1 (ELAM-1) on endothelial cells in EG in humans. | No outcome of interest |
| Patters, 1989 | Assessment of complement cleavage in gingival fluid during EG in man. | No outcome of interest |
| Patters, 1989 | The lymphoproliferative response during human EG. | No full text available |
| Payne, 1975 | Histopathologic features of the initial and early stages of EG in man. | No full text available |
| Persson, 1990 | Relationship between levels of aspartate aminotransferase in gingival crevicular fluid and gingival inflammation. | No outcome of interest |
| Raber-Durlacher, 1993 | EG during pregnancy and post-partum: immunohistochemical aspects. | Wrong design |
| Raber-Durlacher, 1994 | EG during pregnancy and post-partum: clinical, endocrinological, and microbiological aspects. | Wrong population |
| Robertson, 1972 | Collagenolytic activity in EG. | No full text available |
| Rudiger, 2002 | Dental biofilms at healthy and inflamed gingival margins. | Split mouth |
| Salvi, 2012 | Reversibility of experimental peri-implant mucositis compared with EG in humans. | Split mouth |
| Salvi, 2005 | EG in type 1 diabetics: a controlled clinical and microbiological study. | No outcome of interest |
| Salvi, 2010 | Pro-inflammatory biomarkers during EG in patients with type 1 diabetes mellitus: a proof-of-concept study. | No outcome of interest |
| Scapoli, 2005 | Modulation of clinical expression of plaque-induced gingivitis: interleukin-1 gene cluster polymorphisms. | Split mouth |
| Scott, 2012 | Mapping biological to clinical phenotypes during the development (21 days) and resolution (21 days) of EG. | Split mouth |
| Schenk, 1993 | Levels of salivary IgA antibodies reactive with bacteria from dental plaque are associated with susceptibility to EG. | No outcome of interest |
| Seymour, 1983 | Immunohistological analysis of EG in humans. | Split mouth |
| Seymour, 1988 | EG in humans. A clinical and histologic investigation. | No outcome of interest |
| Slotwinska, 1998 | T-cell interactions with extracellular matrix proteins in periodontal disease. | No outcome of interest |
| Syed, 1978 | Bacteriology of human EG: effect of plaque age. | No outcome of interest |
| Thurre, 1984 | Gingival sulcular leukocytes in periodontitis and in EG in humans. | Split mouth |
| Topoll, 1988 | Phenotypic dynamics of macrophage subpopulations during human EG. | No outcome of interest |
| Tsalikis, 2002 | Crevicular fluid levels of interleukin-1alpha and interleukin-1beta during EG in young and old adults. | No full text available |
| Tynekius-Bratthall, 1985 | Fluctuations in crevicular and salivary anti-*A. viscosus* antibody levels in response to treatment of gingivitis. | No outcome of interest |
| Tzamouranis, 1977 | Increase of extracellular cathepsin D activity in gingival washings during EG in man. | Wrong design |
| Van Dyke, 2005 | Effect of topical cimetidine rinse on gingival crevicular neutrophil leukocyte function. | No real healthy control |
| Wahaidi, 2011 | Endotoxemia and the host systemic response during EG. | No outcome of interest |
| Walsh, 1986 | Oral mucosal Langerhans cells express DR and DQ antigens. | No outcome of interest |
| Wynne, 1986 | In situ demonstration of natural killer (NK) cells in human gingival tissue. | No outcome of interest |
| Zee, 1996 | Predominant cultivable supragingival plaque in Chinese "rapid" and "slow" plaque formers. | No outcome of interest |
| Zee, 1996 | Predominant cultivable microflora of supragingival dental plaque in Chinese individuals. | No outcome of interest |
| Zhang, 2010 | Interferon-gamma promoter hypomethylation and increased expression in chronic periodontitis. | Split mouth |
