# Supplementary material for: Resistance and resilience to experimental gingivitis: a systematic scoping review
Source: BMC Oral Health. 2019 Sep 11;19:212. doi: 10.1186/s12903-019-0889-z (PMC6737651; doi:10.1186/s12903-019-0889-z)
Supplement: Supplementary file 2 — Tabulation of parameters included in the final analysis and their main functions (DOCX 45 kb) [file 12903_2019_889_MOESM2_ESM.docx]

**Additional file 2**

| Parameters | Function | References |
| --- | --- | --- |
| Microbiology | |  |
| *Porphyromonas gingivalis* | Gram-negative rod, associated with periodontal disease | 7 |
| *Treponema denticola* | Gram-negative spirochete, associated with periodontal disease | 7 |
| *Tannerella forsythia* | Gram-negative, associated with periodontal disease | 7 |
| *Peptostreptococcus micros* | Gram-positive, non-spore, associated with periodontal disease | 6 |
| *Streptococcus* spp | Gram-positive, coccus, some species are pathogenic most species are not | 6 |
| *Aggregatibacter actinomycetemcomitans* | Gram-negative, non-motile bacterium, associated with localized aggressive periodontitis | 6 |
| *Campylobacter rectus* | Gram-negative, motile bacillus, pathogen in chronic periodontitis | 6 |
| *Fusobacterium nucleatum* | Gram-negative, plays a role in periodontal disease | 6 |
| Immunology | | |
| Intercellular adhesion molecule (ICAM) | Binding of cells to each other, to endothelial cells or extracellular matrix | 19 |
| IgA | Antigen neutralization | 20 |
| IgG | Antigen neutralization | 21 |
| IgM | Antigen neutralization | 21 |
| IL-1 | Regulates immune and inflammatory reactions, stimulates bone resorption | 18 |
| IL-1β | Lymphocyte activating factor | 15,17,19,26 |
| IL-2 | Regulates white blood cell activities | 19 |
| IL-4 | Anti-inflammatory, macrophage inhibition, Th2 differentiation | 19,26 |
| IL-5 | Stimulates B-cell growth and increases immunoglobulin secretion | 19 |
| IL-6 | Regulator of T- and B-cell growth, acute phase protein production | 15,19,24 |
| IL-8 | Recruitment and activation of neutrophils | 24,26 |
| IL-10 | Anti-inflammatory effect | 19 |
| Lymphocytes | Subtype of white blood cell containing natural killer, T- and B-cells from adaptive immune response | 16 |
| Leukocytes | Group of white blood cells | 22,25 |
| Macrophages | Engulfs and digests substances and microbes | 16 |
| Monocyte chemoattractant protein-1 (MCP-1) | Protein that regulates the migration and infiltration of monocytes/macrophages. | 28 |
| PMNs | Antimicrobial function | 15,22,25 |
| Plasma cells | Secretion of antibodies | 16 |
| Leukotriene B4 (LBT4) | Adhesion and activation of leukocytes, chemoattractant | 17 |
| Prostaglandin E2 | Pro-inflammatory and immunomodulatory effects | 17 |
| Thromboxane B2 (TxB2) | Stimulates activities and aggregation of new platelets | 17 |
| Biochemistry | | |
| Cyclooxygenase-2 | Catalyses acid to Prostaglandin | 13 |
| Cystatin | Inhibition of proteolysis | 10 |
| Cystatin C | Inhibition of proteolysis | 10 |
| Elastase | Cleavage of elastin, collagen, proteoglycans | 14,15 |
| Lactoferrin | Antibacterial, creates iron-limiting environment | 9,11 |
| Transferrin | Antibacterial agent, creation iron-limiting environment | 9,21 |
| Vascular endothelial growth factor (VEGF) | Signal protein to create new blood vessels after injury. | 28 |
