# Supplementary material for: Resistance and resilience to experimental gingivitis: a systematic scoping review
Source: BMC Oral Health. 2019 Sep 11;19:212. doi: 10.1186/s12903-019-0889-z (PMC6737651; doi:10.1186/s12903-019-0889-z)
Supplement: Supplementary file 3 — Summary of the EG trial design of included studies, stratified on the basis of healthy subjects and smokers vs non-smokers (DOCX 41 kb) [file 12903_2019_889_MOESM3_ESM.docx]

**Additional file 3**

1. *All healthy*

| Phase | Duration | Reference |
| --- | --- | --- |
| Pre-trial phase | 21 days | 13,25 |
|  | 14 days | 11,16 |
|  | 12 days | - |
|  | 10 days | 9,12 |
|  | 7 days | 8,17 |
|  | 2 days | 23 |
| Induction phase | 7 days | - |
|  | 10 days | 12,14,28 |
|  | 14 days | 11,13 |
|  | 21 days | 8,9,15,16,18,19,21,22,24,25 |
|  | 28 days | 17 |
| Resolution phase | 7 days | 15,24 |
|  | 10 days | - |
|  | 14 days | 8,9,18,19,28 |
|  | 21 days | 11,25 |

1. *Smokers vs non-smokers*

| Pre-trial phase | Unknown | 10,20 |
| --- | --- | --- |
|  | 21 days | 7 |
|  | 14 days | - |
|  | 12 days | 26 |
|  | 10 days | - |
|  | 7 days | - |
|  | 2 days | - |
| Induction phase | 7 days | - |
|  | 10 days | 26 |
|  | 14 days | 7,10,20 |
|  | 21 days |  |
|  | 28 days | - |
| Resolution phase | 7 days | - |
|  | 10 days | - |
|  | 14 days | 7 |
|  | 21 days | - |
