# Supplementary material for: Resistance and resilience to experimental gingivitis: a systematic scoping review
Source: BMC Oral Health. 2019 Sep 11;19:212. doi: 10.1186/s12903-019-0889-z (PMC6737651; doi:10.1186/s12903-019-0889-z)
Supplement: Supplementary file 4 — Methodological quality and potential risk of bias assessment for the included studies. (DOCX 45 kb) [file 12903_2019_889_MOESM4_ESM.docx]

**Additional file 4**

| Author | Were criteria for inclusion in the sample clearly defined? | Were the study subjects and the setting described in detail? | Was the exposure measured in a valid and reliable way? | Were objective, standard criteria used for measurement of the condition? | Were strategies to deal with confounding factors stated? | Were the outcomes measured in a valid and reliable way? | Was appropriate statistical analysis used? | Total score |
| --- | --- | --- | --- | --- | --- | --- | --- | --- |
| Aboodi, 2015^8^ | YES | YES | YES | YES | NO | YES | ? | 5 |
| Adonogianaki, 1994^9^ | YES | YES | YES | YES | NO | YES | YES | 6 |
| Belstrom^28^ | YES | YES | YES | YES | NO | YES | YES | 6 |
| Brecx, 1987^16^ | NO | YES | YES | YES | NO | YES | YES | 5 |
| Giannopoulou, 2003^26^ | YES | YES | YES | YES | NO | YES | YES | 6 |
| Heasman, 1992^17^ | NO | YES | YES | YES | NO | YES | YES | 5 |
| Kinane, 1992^18^ | YES | YES | YES | YES | NO | YES | YES | 6 |
| Leishman, 2013^19^ | NO | YES | YES | YES | NO | YES | YES | 5 |
| Lie, 1998^6^ | YES | YES | YES | YES | NO | YES | YES | 6 |
| Lie, 2001^10^ | YES | NO | YES | YES | NO | YES | YES | 5 |
| Lie, 2002 | YES | YES | YES | YES | NO | YES | YES | 6 |
| Norman, 1978^21^ | YES | NO | YES | YES | NO | YES | YES | 5 |
| Ozedmir, 2009^11^ | YES | NO | YES | YES | NO | YES | YES | 5 |
| Que, 2004^12^ | YES | YES | YES | YES | NO | YES | YES | 6 |
| Reuland-Bosma, 1987^22^ | NO | NO | YES | YES | NO | YES | YES | 4 |
| Salvi, 2005^7^ | YES | YES | YES | YES | NO | YES | YES | 6 |
| Seemann, 2004^23^ | YES | YES | YES | YES | NO | YES | YES | 6 |
| Siegel, 2007^13^ | YES | NO | YES | YES | NO | YES | YES | 5 |
| Tsalikis, 2010^24^ | YES | YES | YES | YES | NO | YES | YES | 6 |
| Uitto, 1996^14^ | NO | YES | YES | YES | NO | YES | YES | 5 |
| Wahaidi, 2009^25^ | NO | YES | YES | YES | NO | YES | YES | 5 |
| Zhou, 2012^15^ | YES | YES | YES | YES | NO | YES | YES | 6 |
